# Characterizing online crowdfunding campaigns for patients with kidney cancer

**DOI:** 10.1002/cam4.3974

**Published:** 2021-06-08

**Authors:** Hannah S. Thomas, Austin W. Lee, Behnam Nabavizadeh, Nikan K. Namiri, Nizar Hakam, Patrick Martin‐Tuite, Natalie Rios, Anthony Enriquez, Nnenaya A. Mmonu, Andrew J. Cohen, Benjamin N. Breyer

**Affiliations:** ^1^ University of Edinburgh School of Medicine Edinburgh UK; ^2^ Department of Urology University of California‐San Francisco San Francisco CA USA; ^3^ The Brady Urological Institute at Johns Hopkins Baltimore MD USA; ^4^ Department of Biostatistics and Epidemiology University of California‐San Francisco San Francisco CA USA

**Keywords:** crowdfunding, fundraising, GoFundMe, insurance, kidney neoplasm

## Abstract

**Background:**

Cancer patients incur high care costs; however, there is a paucity of literature characterizing unmet financial obligations for patients with urologic cancers. Kidney cancer patients are particularly burdened by costs associated with novel systemic treatments. This study aimed to ascertain the characteristics of GoFundMe® crowdfunding campaigns for patients with kidney cancer, in order to better understand the financial needs of this population.

**Methods:**

We performed a cross‐sectional, quantitative, and qualitative analysis of all kidney cancer GoFundMe® campaigns since 2010. Fundraising metrics such as goal funds and amount raised, were extracted. Eight independent investigators collected patient, disease and campaign‐level variables from campaign stories (κ = 0.72). In addition, we performed a content analysis of campaign narratives spotlighting the primary appeal of the patient's life story.

**Results:**

A total of 486 GoFundMe® kidney cancer campaigns were reviewed. The median goal funds were 10,000USD [IQR = 5000, 20,000] and the median amount raised was 1450USD [IQR = 578, 4050]. Most campaigns were for adult males (53%) and 62% of adults had children. A minority were for pediatric patients (17%). Thirty‐seven percent of adult patients were primary wage earners and 43% reported losing their job or substantially reducing hours due to illness. Twenty‐nine percent reported no insurance or insufficient coverage. Campaigns most frequently sought funds for medical bills (60%), nonmedical bills (27%), and medical travel (23%). Qualitative campaign narratives mostly emphasized patients’ hardship (46.3%) or high moral character (35.2%). Only 8% of campaigns achieved their target funds.

**Conclusions:**

Despite fundraising efforts, patients with kidney cancer face persistent financial barriers, incurring both medical and nonmedical cost burdens. This may be compounded by limited or no insurance. Cancer care providers should be aware of financial constraints placed on kidney cancer patients, and consider how these may impact treatment regimens.

## Graphical abstract

This work spotlights online crowdfunding campaigns for patients with kidney cancer. By elucidating patient and campaign characteristics, we are able to better understand the financial needs of this population.

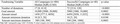

## 1 INTRODUCTION

Crowdfunding is a relatively novel strategy for patients and their families to source funds during times of added financial stress.^1^, ^2^ Approximately eight million Americans have launched a crowdfunding campaign to cover health‐related expenses.^3^ Fifty million Americans report they have contributed to an online, health‐related crowdfunding campaign themselves, which may serve as a lens into national health inequities.^3^, ^4^ The American Society of Clinical Oncology has cited persistent financial barriers for cancer patients, such as the rising cost of cancer medications and increased cost‐sharing burdens.^5^ Along with direct costs, indirect costs such as lost employment time, travel costs, and familial responsibilities, impose cumulative “financial toxicity” on cancer patients.^6^, ^7^ This load has been shown to lead to increased medication non‐adherence, reduced frequency of care visits, and increased symptomatology affecting quality of life.^8^, ^9^ Existing crowdfunding literature demonstrates that cancer patients fundraise online for a variety of different reasons, and differentially benefit those from higher socioeconomic backgrounds.^10^, ^11^ While multiple factors may impact cancer patient's crowdfunding; there is a paucity of data evaluating campaigns for urologic cancers.

Kidney cancer is associated with particularly high financial burden for many reasons, including the rising cost of novel systemic treatments.^12^, ^13^, ^14^ The United States (US) has the second highest incidence of kidney cancer around the world and approximately 73,750 new cases are diagnosed annually.^15^, ^16^ Further, Wilms’ tumor accounts for 5% of pediatric cancer cases nationally.^16^ Kidney cancer causes significant morbidity, accounting for 79.3 disability‐adjusted life years (DALYs) per 100,000 people, and an estimated 15,000 US patients die each year.^16^ It is the sixth most common male cancer in the US, and global rates have risen from 207, 300 incident cases in 1990 to 393, 040 in 2017.^17^ Since 2010, health expenditures due to kidney cancer have also been rising.^18^ The economic burden of metastatic renal cell carcinoma in the US is estimated to be $107–$556 million US Dollars.^19^ With the rising financial burden of kidney cancer care in the US, patients and their families may turn to crowdfunding platforms to cover associated costs.^10^ GoFundMe® is a widely‐known crowdfunding platform where one‐third of campaigns are health‐related, and is most commonly referenced in preceding crowdfunding literature.^10^, ^11^, ^20^

There is a dearth of literature describing the characteristics of kidney cancer patients who seek financial support via online, crowdfunding platforms. This study aimed to ascertain the patient, disease, and campaign‐level characteristics of GoFundMe® campaigns for patients with kidney cancer. We aimed to better understand the financial barriers and current needs of this population.

## 2 MATERIALS AND METHODS

### 2.1 Data collection and study sample selection

**FIGURE 1:**
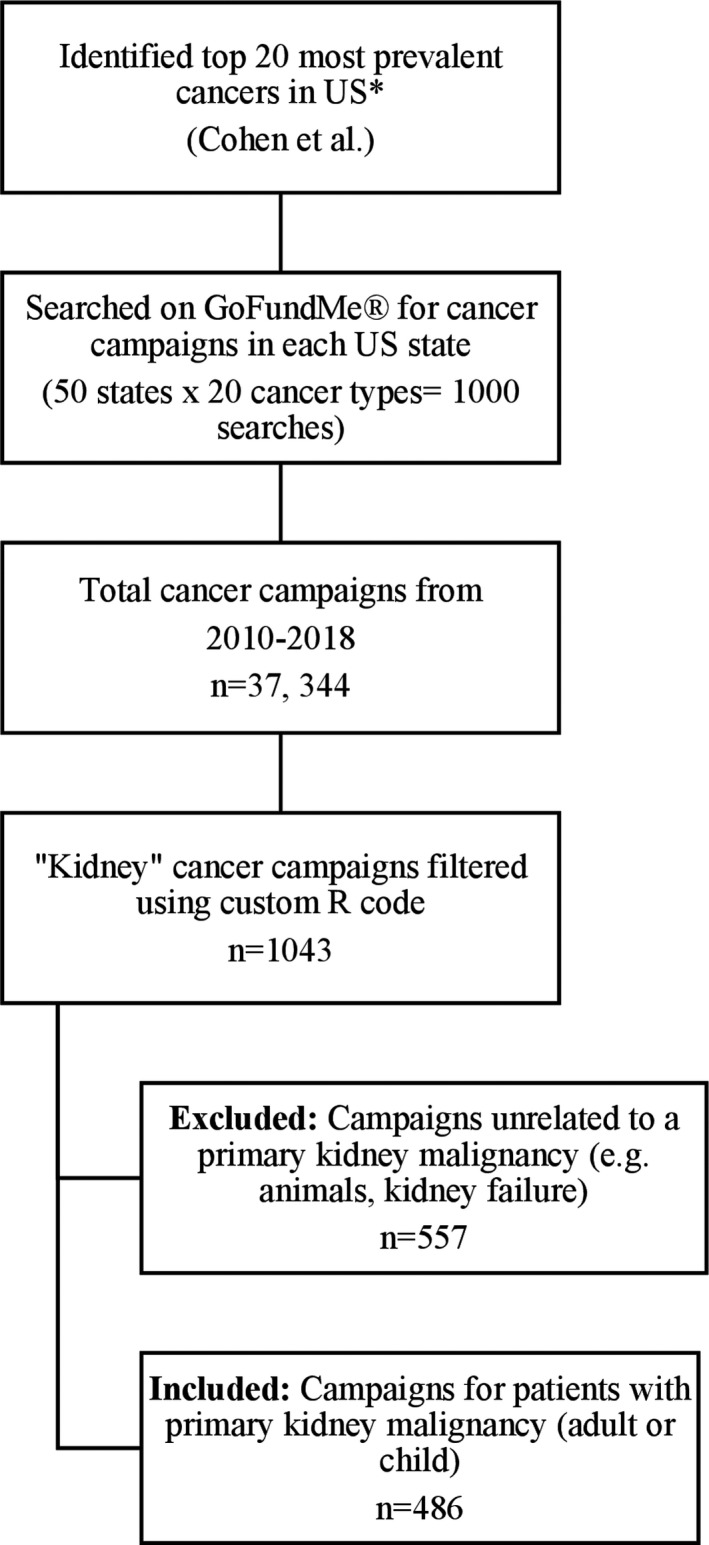
Flowchart of data collection, inclusion and exclusion criteria. *Source: National Cancer Institute

We performed a cross‐sectional, quantitative, and qualitative analysis of GoFundMe® campaigns for patients with kidney cancer. Data collection has been previously described in multispecialty cancer crowdfunding research.^10^ Briefly, Cohen et al. identified the top 20 most prevalent cancers in the US from the National Cancer Institute.^10^, ^21^ Through the GoFundMe® site, they performed 1000 individual searches to identify campaigns for cancer patients in each state (50 states x 20 top cancers = 1000 searches). Any cancer campaign published anywhere in the world since the site's inception in 2010, was included. On October 7, 2018, they used a custom Python code to identify cancer campaigns through web scraping.^10^ Web scraping is an automated process that gathers data from websites to allow analysis.^22^ Data from a total of 37, 344 cancer campaigns was extracted, and several variables, including cancer type, were recorded by Cohen et al (Figure 1).

All cancer campaigns were filtered using a custom RStudio code (Version 1.2.1335) based on cancer type in order to isolate patients with a primary kidney malignancy (*n* = 1043).

### 2.2 Data variables and extraction process

Data were uploaded into Research Electronic Data Capture (REDCap), a secure, online platform for data handling.^23^ Basic campaign features were extracted by HST, including campaign ID, creation date, number of months online, and location. In addition, engagement metrics such as number of photos, hearts (“likes”), shares, updates, comments, and trending status (as classified by GoFundMe® algorithm at the time of data extraction), were extracted as well. Variables pertaining to fundraising efforts, including goal funds, amount raised, minimum, and maximum donations, were also identified. “Successful” campaigns were defined as those which met or surpassed their associated fundraising goal as of Oct 7^th^, 2018.

Two study investigators independently reviewed campaign stories (narratives) (HST, AWL) from 50 randomly selected campaigns. Important characteristics were identified through several rounds of iterative discussion, and a list of variables was created for additional extraction. Demographics, including age (adult = ≥18 years old; child = <18 years old), sex, employment, primary wage status, presence of children, insurance status, and history of military service, were extracted from campaign stories. Patient insurance was classed based on self‐reporting (uninsured, underinsured, insured). Where possible, disease characteristics, such as cancer type, stage, status, and treatment regimen, were also collected. We further defined campaign characteristics including authorship, purpose for requested funds, alternative sources of funding, and reference to religion/spirituality.

For the qualitative data, a content analysis was performed. Prominent themes pertaining to the patient's main narrative were independently identified from 50 campaigns by HST and AWL, both medical students with interests in urology and kidney cancer. After multiple rounds of discussion, a codebook was created with defined a priori categories (patient's hardship, high moral character, contribution to society, other) for patient's life story primary appeal.

Eight study investigators (HST, AWL, BN, NKN, NH, PMT, NR, and AE) were trained via a one‐hour online session on how to perform the quantitative data extraction, and qualitative coding. Afterward, they all independently extracted data from a trial of 15 kidney cancer campaigns and interrater reliability was examined (κ = 0.72). At this stage, repetitive discrepancies in data collection were openly discussed with all investigators and additional training was provided.

Subsequently, each investigator extracted all patient, disease and campaign‐level variables, from a designated number of kidney cancer campaigns. Patient stories were coded into one of the above qualitative categories and exemplar excerpts were collected from each campaign. Due to the volume of campaigns, multiple coders did not code the same campaign. All data extraction and coding was performed in REDCap.^23^

During the data extraction process, investigators confirmed the inclusion of campaigns that met the eligibility criteria of a primary kidney malignancy (*n* = 486). Pediatric kidney malignancies, such as Wilms’ tumor, were included. Excluded campaigns were ​mostly those related to animals or kidney failure secondary to a non‐cancer‐related illness.

### 2.3 Statistical analysis

Data were exported from the REDCap platform and descriptive statistics were performed using RStudio (Version 1.2.1335). Campaigns were stratified into “all campaigns” and those that were “successful,” as defined above. The denominators for both categories reflect the total number of campaigns, rather than just the campaigns that mentioned the variable. This is important for a sub select of our variables. For example, campaigns which did not mention travel out of state/country for treatment were assumed to not have travelled out of state/country or did not feel it was meaningful enough to include in the campaign narrative. The denominator is therefore reported as the total number of campaigns, regardless. Bivariate analysis was performed to explore differences between patient, disease, and campaign‐level characteristics and campaign success. We used chi‐square or Fisher's exact test to compare categorical variables for differences in proportions. *p*‐values < 0.05 were statistically significant.

### 2.4 Ethics

The institutional review board at the University of California, San Francisco deemed this study exempt because all data were publicly available and de‐identified.

## 3 RESULTS

### 3.1 General and fundraising characteristics

A total of 1043 kidney cancer campaigns were initially filtered from Cohen et al's cohort of GoFundMe® cancer campaigns.^10^ Of these, 486 met the above inclusion criteria and were included in the final analysis. Campaigns were posted online for a median of 22 months [Interquartile Range (IQR) = 8.3, 34], shared 154 times [IQR = 65, 350.8] and ‘hearted’ 19 times [IQR = 9, 43]. The median number of comments, updates, and pictures per campaign were 1 [IQR = 0,4], 2 [IQR = 0, 4], and 1 [IQR = 1,1], respectively. Campaigns were located in the West (24.3%, 118/486), Midwest (22%, 107/486), Southeast (20.2%, 98/486), Southwest (16.7%, 81/486), and Northeast (10.3%, 50/486), regions of the US. Elsewhere, campaigns were located in Canada (2.5%, 12/486) and Australia (0.6%, 3/486). A minority of campaigns were trending on the site (3.5%, 17/486).

The median number of donations per campaign was 17 [IQR = 8, 41.8] (Table 1). In general, campaigns were seeking 10,000 US dollars (USD) [IQR = 5000, 20,000], and raised 1450 USD [IQR = 577.5, 4050]. The median minimum and maximum donations were 10 USD [IQR=8.5, 20] and 300 USD [IQR = 100, 500], respectively. Contrastingly, successful campaigns had a median 72 donations per campaign [IQR = 23.8, 120], 5000USD goal amount [IQR = 1000, 10,000] and 6450USD amount raised [IQR=2030, 11, 032].

**TABLE 1:** Fundraising characteristics of online GoFundMe® campaigns for patients with kidney cancer. *n* = 486

Fundraising Variable	All Campaigns (*n* = 486) Median [IQR] (USD)	Successful Campaigns (*n* = 40) Median [IQR] (USD)	*p*‐value
Number of donations	17 [8, 41.8]	72 [23.8, 120]	<0.0001
Goal amount	10,000 [5000, 20,000]	5000 [1000, 10,000]	0.0002
Amount raised	1450 [577.5, 4050]	6450 [2030, 11,032]	<0.0001
Amount minimum donation	10 [8.5, 20]	10 [5, 20]	0.0100
Amount maximum donation	300 [100, 500]	500 [288, 1000]	0.0003

Note

*p*−values compare rows with campaign success (unsuccessful vs. successful).

Abbreviations: IQR, interquartile range; USD, US Dollar.

### 3.2 Patient, disease, and campaign‐level characteristics

The majority of kidney cancer campaigns were for adult male patients (53.3%, 259/486) (Table 2). Seventeen percent of campaigns were for pediatric patients (82/486). Most adult patients had children (61.9%, 249/402). Approximately one third of adult patient campaigns (34.3%, 138/402) mentioned that the patient was employed; however, 43.3% (174/402) reported a job loss or reduction in hours due to illness. Thirty‐seven percent (150/402) of adult patients were primary wage earners within their family. Twenty‐nine percent of campaigns mentioned that the patient had no insurance (10.7%, 52/486) or was under‐covered (18.1%, 88/486). Finally, 10.2% (41/402) of campaigns mentioned that the patient was a veteran or in the military.

**TABLE 2:** Patient, disease, and campaign‐level characteristics of online GoFundMe® campaigns for patients with kidney cancer. *n* = 486

Characteristic	All Campaigns *n* = 486 *n* (%)	Successful Campaigns *n* = 40 *n* (%)	*p*‐value
Patient^a^			
Patient age			
Adult	402 (82.7)	30 (75)	0.313
Child	82 (16.9)	10 (25)	
Patient sex			
Male	298 (61.3)	27 (67.5)	0.436
Female	174 (35.8)	13 (32.5)	
Unknown	14 (2.9)	0 (0) 0 (0)	
Employment status			
Adult, Yes	138 (34.3)	11 (36.7)	0.644
Parent of child, Yes	29 (35.4)	5 (50)	0.217
Loss of job/reduction of hours due to illness			
Adult, Yes	174 (43.3)	12 (40)	0.526
Parent of child, Yes	24 (29.3)	5 (50)	0.147
Patient is primary wage earner	150 (37.3)	14 (46.7)	0.447
Patient has children	249 (61.9)	17 (56.7)	0.484
Patient has children <18y/o	77 (19.2)	7 (23.3)	0.179
Insurance status			
Covered	20 (4.1)	1 (2.5)	0.695
Under‐covered	88 (18.1)	5 (12.5)	
No insurance	52 (10.7)	3 (7.5)	
Unknown	326 (67.1)	31 (77.5)	
Patient is in military or a veteran	41 (10.2)	2 (6.7)	0.330
Disease			
Cancer type			
Renal cell carcinoma	98 (20.2)	11 (27.5)	0.219
Wilms’ tumor	41 (8.4)	5 (12.5)	
Other	16 (3.3)	2 (5)	
Unknown	331 (68.1)	22 (55) 22 (55)	
Cancer stage			
Early (I)	4 (0.8)	0 (0)	0.371
Mid (II or III)	24 (4.9)	4 (10)	
Late (IV+)	136 (28)	14 (35)	
Unknown	317 (65.2)	22 (55)	
Cancer status			
Active treatment	259 (53.3)	17 (42.5)	0.097
Metastasis	64 (13.2)	10 (25)	
Remission/cure	56 (11.5)	3 (7.5)	
Hospice/end of life	23 (4.7)	2 (5) 2 (5)	
Death	36 (7.4)	6 (15)	
Unknown	47 (9.7)	2 (5)	
Metastases, yes			0.889
Bone	39 (8)	4 (10)	
Lymph Nodes	23 (4.7)	2 (5)	
Brain	22 (4.5)	2 (5)	
Lung	16 (3.3)	1 (2.5)	
Adrenal	8 (1.6)	1 (2.5)	
Liver	5 (1)	2 (5)	
Pancreas	3 (0.6)	2 (5)	
Colon	2 (0.4)	0 (0)	
Ovary	2 (0.4)	0 (0)	
Uterus	1 (0.2)	0 (0)	
None	141 (35.2)	10 (25)	
Other	26 (5.3)	3 (7.5)	
Unknown	236 (48.6)	19 (47.5)	
Travel out of state/country for treatment, yes	57 (11.7)	7 (17.5)	0.091
History of misdiagnosis or delayed diagnosis, yes	24 (4.9)	3 (7.5)	0.209
Past treatment, yes			0.574
Surgery	222 (45.7)	15 (37.5)	
Chemotherapy	79 (16.3	7 (17.5)	
Radiation	48 (9.9)	3 (7.5)	
Experimental treatment	10 (2.1)	1 (2.5)	
Immunotherapy	8 (1.6)	2 (5)	
Transplant	3 (0.6)	2 (5)	
Stem cell treatment	1 (0.2)	0 (0)	
Bone marrow transplant	0 (0)	0 (0)	
None	104 (21.4)	10 (25)	
Unknown	115 (23.7)	11 (27.5)	
Future treatment, yes			0.603
Surgery	125 (25.7)	12 (30)	
Chemotherapy	90 (18.5)	6 (15)	
Radiation	51 (10.5)	4 (10)	
Experimental treatment	16 (3.3)	1 (2.5)	
Transplant	12 (2.5)	1 (2.5)	
Immunotherapy	9 (1.9)	1 (2.5)	
Stem cell treatment	2 (0.4)	1 (2.5)	
Bone marrow transplant	1 (0.2)	0 (0)	
None	89 (17.1)	5 (12.5)	
Unknown	183 (37.7)	17 (42.5)	
Campaign			
Author of GoFundMe® campaign			
Friend	140 (28.8)	13 (32.5)	0.205
Other family	72 (14.8)	4 (10)	
Child	70 (14.4)	5 (12.5)	
Self	64 (13.2)	1 (2.5)	
Spouse	26 (5.3)	3 (7.5)	
Parent	15 (3.1)	2 (5)	
Unknown	97 (20)	12 (30)	
Fundraising out with GoFundMe®^b^	39 (8)	3 (7.5)	0.597
Reference to religion or spirituality	206 (42.4)	22 (55)	0.092

^a^ Proportions were tabulated based on either the number of total patients (*n* = 486), the number of adult patients (*n* = 402) or the number of child patients (*n* = 82), depending on relevant context. For example, for employment status of the parent of the child, the denominator = 82.

^b^ Referring to any other form of fundraising for patient's expenses, as mentioned in the campaign story (e.g. community barbeque, gala, etc…). *p*‐values compare rows with campaign success (unsuccessful vs. successful).

We were largely unable to ascertain which kidney cancer type was present from campaign stories (68.1%, 331/486); however, 20.2% were reported as renal cell carcinoma (RCC) (98/486) (Table 2). Most campaigns reported that patients were in a late stage (28%, 136/486) and undergoing active treatment (53.3%, 259/486). In cases of metastasis, cancer had largely spread to bone (8%, 39/486), lymph nodes (4.7%, 23/486), and brain (4.5%, 22/486). Twelve percent of patients travelled out of state or country for treatment (57/486). Prior to their campaign launch, many patients had already undergone surgery (45.7%, 222/486). Campaigns subsequently mentioned additional surgery (25.7%, 125/486), chemotherapy (18.5%, 90/486), and radiotherapy (10.5%, 51/486) as part of future treatment.

**FIGURE 2:**
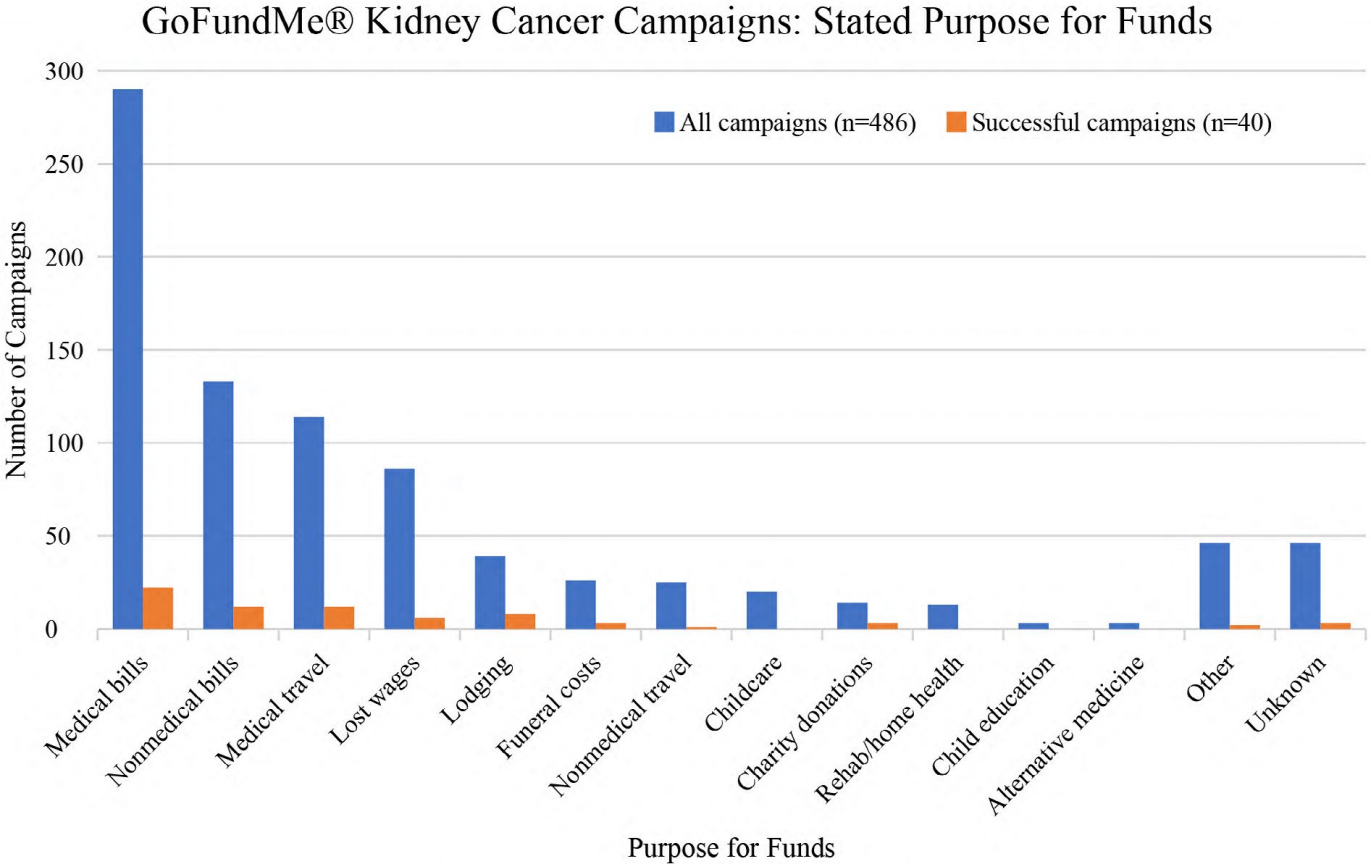
Stated purpose for requested funds among GoFundMe® campaigns for patients with kidney cancer

Campaigns were primarily authored by friends of the patient (28.8%, 140/486), other family (14.8%, 72/486), or the patient's child (14.4%, 70/486) (Table 2). The majority of campaigns mentioned that funds contributed towards medical bills (59.7%, 290/486) (Figure 2). Nonmedical bills (27.4%, 133/486), medical travel (23.5%, 114/486), and lost wages (17.7%, 86/486) accounted for much of the remaining funds. Eight percent of campaigns mentioned alternative sources of fundraising out with GoFundMe® (39/486). Approximately 42% of campaigns referenced religion or spirituality (206/486).

Finally, 8.2% of campaigns were deemed “successful” (Table 2). Successful campaigns were mostly for adult (75%, 30/40), male (67.5%, 27/40) patients (Table 2). The majority of adults had children (56.7% 17/30) and near half described themselves as primary wage earners (46.7%, 14/30). Twenty percent of successful campaigns reported no or insufficient insurance (8/40). Patients most commonly sought funds for medical bills (55%, 22/40), nonmedical bills, and medical travel (30%, 12/40) and lodging (20%, 8/40). The majority of successful campaigns referenced religion or spirituality in their campaign story (55%, 22/40).

### 3.3 Qualitative review: patient's life story primary appeal

Study investigators reviewed campaign stories to evaluate the patient's life story (Table 3). The life story refers to the general sentiment and/or rousing narrative employed by the campaign author to appeal for financial support. Most campaign stories centered around the patient's hardships/ disheartening circumstances (46.3%, 225/486). Anecdotally, for pediatric patients, campaigns often expressed saddened concern for how young the child was. Campaign authors also frequently referenced the patient's high moral character (35.2%, 171/486), praising intrinsic traits such as kindness and compassion and societal archetypes such as devotion to family. Finally, a minority of campaign narratives centered around describing the patient's contribution to society (11.9%, 58/486). This was often conveyed as a patient's particular talent, generosity, or service to community such as military duty. Few campaign primary appeals were captured as “Other.”

**TABLE 3:** Qualitative analysis of patient's life story primary appeal

Theme	Definition	Frequency *n* (%)	Exemplar excerpts
Hardship/disheartening circumstances	Campaign story emphasized the patient's hardships/disheartening circumstances, in an appeal to gather fundraising support (e.g. how young the patient is, many bad things happened back‐to‐back that led to present situation)	225 (46.3)	*“But we found out that when the recession hit back in 2008, revenue slowly started to decline at the restaurant…. Also, during this same period, with housing values declining, like many Americans, their home mortgage was significantly more than the value of their house. We thought that in a few years, they would bounce‐back, which hasn't happened only to be compounded by a series of health issues…”* *“[Patient name] is only three years old and to have a fighting chance she will need to undergo further treatment…[patient name] also has a beautiful little brother*, *who at the tender age of 18 months wants nothing more than for his big sister to get better so they can play again*.”
High moral character	Campaign story emphasized positive characteristics and society‐based values that the patient possesses, in an appeal to gather fundraising support (e.g. compassionate, kind, giving person, family man/woman)	171 (35.2)	*“Many know my dad for his sense of humor, his kind smile, and how he is astoundingly and uncannily knowledgeable on just about any random topic you can think of. He is the hardest‐working man I know. He can rebuild a motor, restore a car, cook you the best dinner you've ever had, dazzle you with his storytelling skills, and crack an epic joke (few know he can sing too).”* *“[Patient name] was the icon of the restaurant, he was the one that made people feel good and enjoy their meal and the atmosphere! He had a zest for life and joy for everyone! Not one customer had a meal there that didn't get enjoy their meal and feel good when they left.”*
Contribution to society	Campaign story emphasized significant contribution made to community/society by the patient, in an appeal to gather fundraising support (e.g. exquisite talent, veteran/military service, community worker)	58 (11.9)	*“[Patient name] has devoted her entire life to nursing and the care of others. She honorably served her country as a nurse in the US Armed Forces. Being recognized for her dedicated service she obtained the rank Major in the US Army.”* *“[Patient name] has been a very visible and respected member in the community for decades. Those who know [him], know he is a selfless person who does so much to support and promote all of the great accomplishments by our student‐athletes...”*
Other	Campaign story emphasized something not captured in the above categories	30 (6.2)	*“The purpose of this Go Fund Me account is to raise financial support to fight what is not mine which is kidney cancer (renal cell carcinoma) and provide for the costs of medical bills. Beyond money I covet your prayers and encouragement the most!”*

## 4 DISCUSSION

This study uniquely described the characteristics of online crowdfunding campaigns for patients with kidney cancer. GoFundMe® kidney cancer campaigns were most frequently utilized for adult, male patients, of whom were in a late disease stage and undergoing active treatment. A high proportion of patients lost their job or reduced their working hours due to illness. In parallel, one quarter reported insufficient or no insurance. Written by friends and family, crowdfunding campaigns for kidney cancer mostly asked for funds to cover medical bills, medical travel, and nonmedical bills such as lost wages. However, per campaign, the median deficit was 8550 USD, meaning patients frequently received less than asked for and only 8% of campaigns were “successful.” There were key descriptive narratives encouraging readers to donate to respective campaigns, including emphasis on patient hardships and high moral character.

Crowdfunding platforms facilitate the opportunity for alignment of patient and donor priorities, ultimately permitting strategic fundraising towards medical expenses.^2^ Previous work by Cohen et al. highlighted a significant database of crowdfunding campaigns for patients with prevalent cancer types.^10^ When compared with our kidney cancer‐specific analysis, the findings from both studies appeared to support one another. In both, patients sought financial assistance toward medical bills, medical travel, and nonmedical bills.^10^ For kidney cancer patients, this may be reflective of literature demonstrating the rising costs of systemic therapies and consequent financial toxicity impacting management decisions.^12^ While medical costs remain significant, our results underscored less‐commonly considered indirect cost burdens such as lost wages and lodging as well. Additional research has found that patients also crowdfund in order to cover costs of experimental and “scientifically unsupported” treatments to achieve therapeutic success and pain reduction.^24^, ^25^, ^26^ However, when compared with more prevalent cancers such as breast, colon and lung, patients with kidney cancer less commonly sought funds for complementary and alternative medicine‐ this was echoed by our results.^26^ Instead, patients chiefly called for treatment‐related financial assistance for more widely‐accepted therapies such as nephrectomy, radiotherapy, and chemotherapy.^27^ Finally, when compared with Cohen et al.’s multiorgan cancer cohort, it may be notable to mention that a higher proportion of kidney cancer patients reported themselves as the primary wage earner and had subsequent loss of job/reduction of hours due to illness.

In our study, we found that 486 patients with kidney cancer requested financial assistance through GoFundMe® from 2010 to 2018. However, patients with kidney cancer appeared to receive only a small proportion of the total funds sought from their campaign, leaving many with apparent insufficient coverage of medical and nonmedical costs. This may be harmful for patients and their families, alike. Research has shown that financial barriers can result in reduced or delayed access to targeted drug therapies among patients with metastatic RCC.^28^ Moreover, despite the advancement of cost‐reduction strategies for children undergoing treatment for Wilms’ tumor, families are often still left indebted.^29^ Additional insight from surrounding analyses of GoFundMe® campaigns, suggest that financial cancer burdens are greater for patients living in rural communities than those from urban regions.^30^ Rural cancer patients report higher rates of unemployment and no insurance, along with clinical variables such as metastatic cancer rate.^30^ In our cohort, the proportion of patients who travelled out of state/country for treatment (11%) may yield insight into the financial influence of proximity to specialized kidney cancer care. Overall, healthcare providers should be vigilant in exploring and addressing financial vulnerabilities for rural patients, during clinical encounters.

Furthermore, within both our cohort and previously‐published multispecialty cancer work, approximately one quarter of campaigns mentioned that patients were uninsured or under covered.^10^ In a cohort of high‐income countries, crowdfunding campaigns for routine treatment expenses were more commonly found in the US.^20^ Conceptually, crowdfunding may be a proxy for health inequities, pushing users toward reliance on private financing opportunities as health systems do little to support mounting care costs.^4^ As crowdfunding methods gain popularity, this may represent a direct reflection of the current healthcare crisis.^4^, ^31^, ^32^ Comprehensive social health insurance is one such strategy that has been shown to facilitate improved access to cancer care, and benefits are greatest for those most disadvantaged.^33^

This study is limited by the depth and breadth of data gathered from online GoFundMe® cancer campaign submissions. Investigators were reliant on self‐reported patient narratives and were unable to confirm the accuracy, and in some cases, the full context of a statement. Denominators are presented as the total numbers of campaigns as with this methodology, it was impossible to ask campaign authors/patients about each specific variable and vary the denominator accordingly. In addition, it is challenging to draw differential conclusions regarding the characteristics and success of GoFundMe® campaigns for kidney cancer patients, in particular, as this study reported no comparator group. Finally, it is largely unclear whether the features of this cohort of GoFundMe® patients were generalizable to those of the general population. However, we believe this is an important step in capturing the habits of this important demographic group and future work exploring crowdfunding for other urologic cancers.

## 5 CONCLUSIONS

Among a cohort of crowdfunding campaigns for patients with kidney cancer, patients faced financial barriers to care, including the loss/reduction of hours at work. As a result, financial assistance was frequently sought for medical bills, medical travel, and nonmedical bills. Despite a variety of patient narratives, campaigns generally did not achieve their target funds, leaving patients with persistent debt. In the absence of successful compensation through crowdfunding, clinicians must continue to advocate for strategies to reduce the financial burden of cancer care for their patients. Collaborative efforts with multispecialty healthcare professionals, including urologists and oncologists, economists, and policymakers should inform future strategies to mitigate financial health inequities for patients with kidney cancer.

## CONFLICT OF INTEREST

The authors declares no conflict of interest.
